# Locus-Specific Biochemical Epigenetics/Chromatin Biochemistry by Insertional Chromatin Immunoprecipitation

**DOI:** 10.1155/2013/913273

**Published:** 2013-01-10

**Authors:** Toshitsugu Fujita, Hodaka Fujii

**Affiliations:** Combined Program on Microbiology and Immunology, Research Institute for Microbial Diseases, Osaka University, 3-1 Yamadaoka, Suita, Osaka 565-0871, Japan

## Abstract

Comprehensive understanding of regulation mechanisms of biological phenomena mediated by functions of genomic DNA requires identification of molecules bound to genomic regions of interest *in vivo*. However, nonbiased methods to identify molecules bound to specific genomic loci *in vivo* are limited. To perform biochemical and molecular biological analysis of specific genomic regions, we developed the insertional chromatin immunoprecipitation (iChIP) technology to purify the genomic regions of interest. We applied iChIP to direct identification of components of insulator complexes, which function as boundaries of chromatin domain, showing that it is feasible to directly identify proteins and RNA bound to a specific genomic region *in vivo* by using iChIP. In addition, recently, we succeeded in identifying proteins and genomic regions interacting with a single copy endogenous locus. In this paper, we will discuss the application of iChIP to epigenetics and chromatin research.

## 1. Introduction

Detailed biochemical and molecular biological analysis of chromatin domains is critical for understanding mechanisms of genetic and epigenetic regulation of gene expression, hetero- and euchromatinization, X-chromosome inactivation, genomic imprinting, and other important biological phenomena [1]. However, biochemical nature of chromatin domains is poorly understood. This is mainly because methods for performing biochemical and molecular biological analysis of chromatin structure are limited [2–8].

Identification of regulatory regions of gene expression has been extensively attempted in the last several decades. Conventionally, these analyses have been performed by using artificial methods such as reporter assay [9] and *in silico* identification of genomic regions conserved among species [10]. More recently, enhancer-specific modifications are being used to identify enhancer regions in the genome (see review [11]). However, although these approaches have been successful for relatively easy targets such as immediate early genes, it has been shown that they could produce artifactual results in many circumstances. In fact, deletion studies of candidate regulatory endogenous genomic regions have shown that the candidate regions identified by using these conventional methods could often be dispensable for expression of the genes of interest. Furthermore, these approaches cannot be used when regulatory genomic regions are far from regulated loci, for example, on other chromosomes. In fact, long-range interaction including interchromosomal interaction has been suggested to play important roles in regulation of gene expression and other biological phenomena [12]. In this regard, it has been shown that such regulatory regions have physical contact with the regulated loci, forming a loop [13, 14]. This led to the idea of identification of regulatory genomic regions by detecting genomic regions interacting with the genomic region of interest. Thus, development of methods to identify intra- and interchromosomal interaction is vital for the advancement of the field.

Identification of molecules such as proteins and RNAs interacting with specific genomic regions is also essential for understanding of epigenetic regulation and chromatin biology. Conventionally, molecules interacting with a specific genomic region have been identified using artificial approaches including affinity purification, yeast one-hybrid, electrophoretic mobility shift assay (EMSA), and others [15]. Although these approaches are successful in some cases, especially for the analyses of easier targets such as immediate early responses, they can be very problematic. For example, experimental conditions in these artificial approaches are far from physiological, causing artifactual or misleading results. Therefore, researchers need to verify if the detected interaction is physiological using other *in vivo* approaches. This requires a lot of efforts and takes long time, often more than 10 years. These problems have delayed the advancement of the field. Therefore, development of technologies that detect molecular interaction on the genome *in vivo* is absolutely required.

In this paper, we will first discuss conventional techniques to analyze the molecular interaction on the genome *in vivo*. Subsequently, we will discuss insertional chromatin immunoprecipitation (iChIP) we developed for the locus-specific biochemical epigenetics/chromatin biochemistry and its application.

## 2. Methods to Analyze Molecular Interaction *In Vivo*

Several methods have been devised to analyze molecular interaction with specific genomic regions *in vivo*.

### 2.1. Chromatin Immunoprecipitation (ChIP)

**Figure 1:**
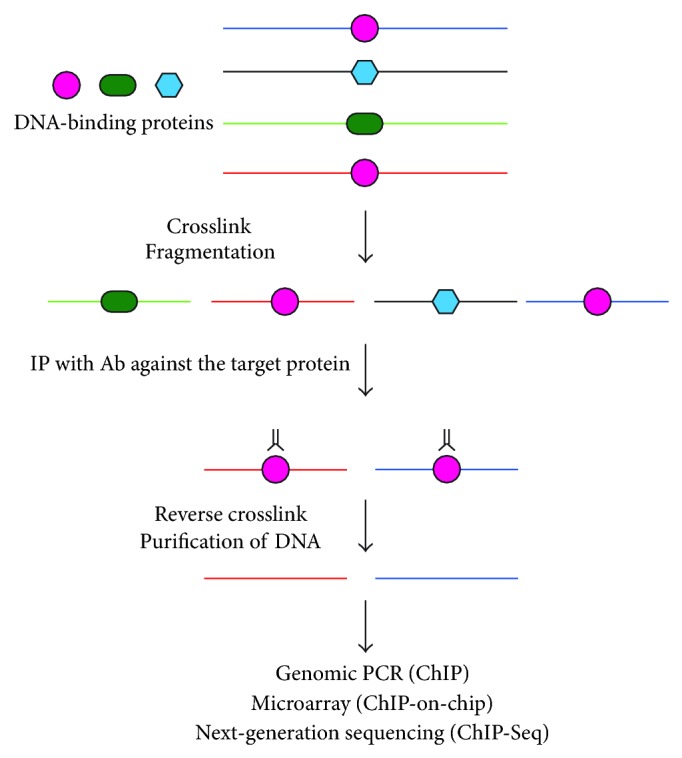
Scheme of ChIP. In ChIP, molecular interaction can be preserved by crosslinking with formaldehyde or other crosslinkers. Subsequently, chromatin is fragmented by sonication or digestion with endonucleases. Immunoprecipitation with antibodies against DNA-binding proteins of interest is performed to isolate genomic regions bound by the DNA-binding proteins.

ChIP was developed in 1988 [16] and has played instrumental roles in detection of molecular interaction in the genome *in vivo*. In ChIP, molecular interaction can be preserved by crosslinking with formaldehyde or other crosslinkers. Subsequently, chromatin is fragmented by sonication or digestion with endonucleases. Immunoprecipitation with antibodies against DNA-binding proteins of interest is performed to isolate genomic regions bound by the DNA-binding proteins (Figure 1). ChIP has been used to identify *in vivo* binidng of transcription factors and other chromatin-associated factors. Recently, by combining with DNA microarray analysis (ChIP-on-chip) or next-generation sequencing (ChIP-Seq), ChIP has been used for genome-wide search for target sequences bound by a given DNA-binding protein [17].

Although ChIP is a very powerful technique and revolutionized epigenetics and chromatin research, it has some limitations. For example, although ChIP is essential to identify genomic loci to which a given protein binds, it cannot be used to identify unknown proteins binding to genomic loci of interest.

### 2.2. Imaging Analyses

Imaging techniques have been widely used to examine molecular interaction with specific genomic regions [18, 19]. Fluorescent *in situ* hybridization (FISH) is used to visualize specific genomic loci. Proteins and RNA interacting with a genomic locus of interest are detected by immunofluorescence and *in situ* hybridization, respectively.

They have, however, certain limitations: (i) resolution is low; that is, even if FISH and protein signals look co-localized, it does not necessarily mean the protein is in that locus. The protein can be localized far from that locus. It cannot be judged by the imaging methods. (ii) Nonbiased search for interacting proteins and RNA is not feasible. Before colocalization is examined by imaging techniques, candidate proteins and RNA should be identified by other methods.

### 2.3. Chromosome Conformation Capture (3C) and Its Derivatives

**Figure 2:**
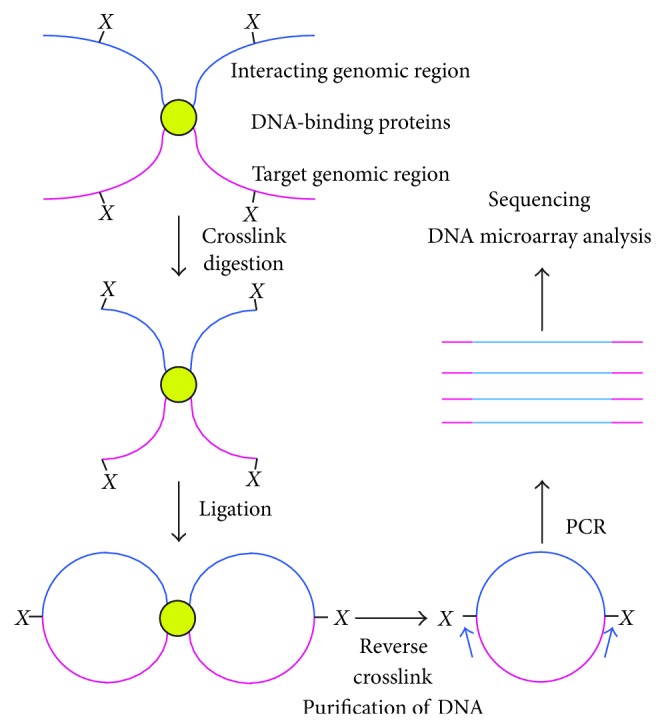
Scheme of 3C-based identification of interacting genomic regions. In 3C, molecular interaction is maintained by crosslinking with formaldehyde before digesting with a restriction enzyme(s). After ligation of DNA ends in the same complex, crosslink is reversed and DNA is purified. Interaction of genomic loci is detected by PCR using locus-specific primers or microarray/NGS.

3C was developed in 2002 to examine genome-genome interaction [20]. In 3C, molecular interaction is maintained by crosslinking with formaldehyde before digesting with a restriction enzyme(s). After ligation of DNA ends in the same complex, proteins and RNA are removed by phenol/chloroform extraction to purify DNA. Interaction of genomic loci is detected by PCR using locus-specific primers (Figure 2). By using 3C, interaction of genomic loci including interferon-*γ* and IL-4 loci [12] and odorant receptor loci and its regulatory locus [21] has been demonstrated. In addition, unknown interaction can be detected by PCR using primers annealing with both ends of target genomic fragments (4C and 5C) [22] and HiC [23] (see the review [24] for details on 3C derivatives). 3C has been widely used these days for the genome-wide genome interaction analysis that is one of important categories of epigenomics.

However, 3C-based approaches have some intrinsic drawbacks. (i) 3C-based methods detect only genome-genome interaction. Information on neither interacting proteins nor RNA can be obtained. (ii) 3C-based methods require enzymatic reactions including digestion with restriction enzymes and ligation of crosslinked chromatin. Especially, difficulty in complete digestion of crosslinked chromatin can cause detection of artifactual interaction. In fact, it has been shown that interaction detected by 3C does not necessarily correspond to that detected by imaging approach [25]. (iii) Allele-specific analysis is very difficult, if not impossible; that is, 3C-based methods are not able to detect allele-specific interaction. This problem would make it difficult to apply 3C-based methods to analysis of genomic imprinting for example.

### 2.4. Proteomics of Isolated Chromatin (PICh)

**Figure 3:**
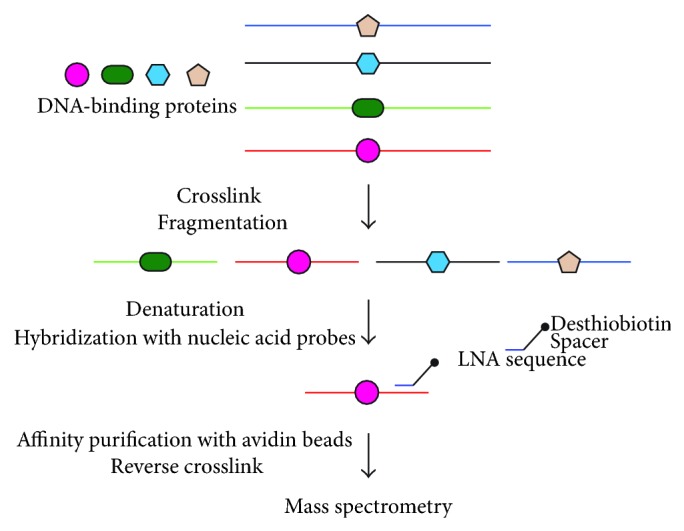
Scheme of PICh. PICh utilizes specific biotinylated nucleic acid probes such as locked nucleic acids (LNAs) that hybridize target genomic regions and isolates the regions using streptavidin beads to analyze interacting proteins.

PICh is a novel technique to isolate specific genomic regions retaining molecular interaction [8]. PICh utilizes specific biotinylated nucleic acid probes such as locked nucleic acids (LNAs) that hybridize target genomic regions and isolates the regions using streptavidin beads to analyze interacting proteins (Figure 3). It has been shown that human telomeres can be successfully isolated to identify interacting proteins [8]. PICh would be especially useful to isolate genomic regions containing multiple repeats.

On the other hand, PICh also has its intrinsic problems. (i) It would be difficult to apply PICh to isolation of low-copy number genomic loci. Since PICh requires partial denaturing of crosslinked target genomic loci, it would be very difficult to efficiently hybridize probes to low-copy number loci, making it very difficult to obtain sufficient amounts of those loci for biochemical analysis. (ii) When genomic regions containing repeated sequences are isolated by PICh, the isolated complexes are heterogeneous, that is, a mixture of different loci. For example, isolated telomeres by PICh are mixtures of telomeres of distinct chromosomes. It is not feasible to isolate telomeres of a certain chromosome (e.g., chromosome 1). (iii) Allele-specific analysis is not feasible by PICh.

## 3. Insertional Chromatin Immunoprecipitation (iChIP)

### 3.1. Principle of iChIP

**Figure 4:**
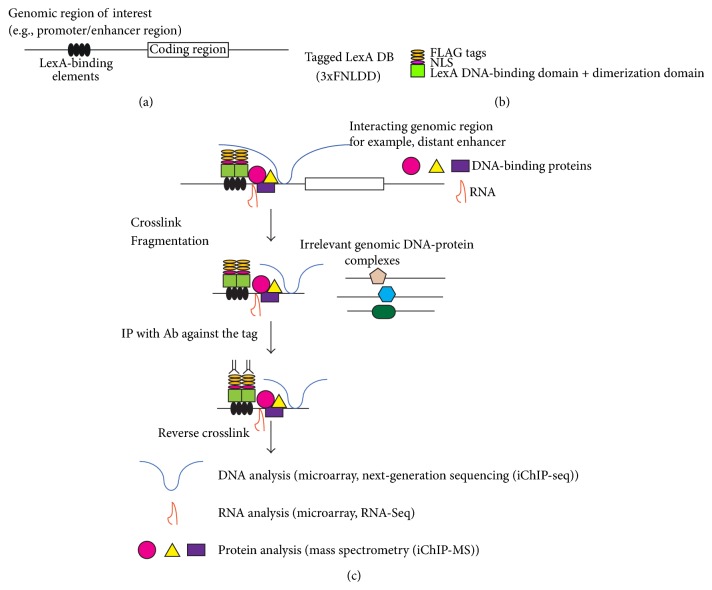
Scheme of insertional chromatin immunoprecipitation (iChIP). The system consists of a locus of interest (e.g., a promoter, an enhancer, and an silencer of a gene) linked to LexA-binding elements (LexA BE) (a), and FLAG-tagged, nuclear localization signal (NLS)-fused LexA DNA-binding domain (DB) (3xFNLDD) (b). LexA-binding sites are knocked-in in the genomic locus of interest in cells expressing the tagged LexA DB. Alternatively, cells expressing the tagged LexA DB are transiently or stably transfected with the transgene tagged with LexA BE. These cells are crosslinked with formaldehyde or other crosslinkers, if necessary, and lysed. Then, DNA is fragmented by sonication or other methods. Subsequently, the LexA-tagged genomic region is immunoprecipitated with an anti-FLAG antibody, and crosslink is reversed when a crosslinker is used. Molecules (DNA, RNA, proteins, and others) associated with the LexA-tagged genomic regions are characterized (c).

To perform biochemical analyses of specific genomic regions retaining molecular interaction, we developed insertional chromatin immunoprecipitation (iChIP) [26]. The scheme of iChIP is as follows (Figure 4). (i) A repeat of the recognition sequence of an exogenous DNA-binding protein such as LexA is inserted into the genomic region of interest in the cell to be analyzed (Figure 4(a)). (ii) The DNA-binding domain (DB) of the exogenous DNA-binding protein is fused with a tag(s) and a nuclear localization signal (NLS)(s) and expressed into the cell to be analyzed (Figure 4(b)). (iii) The resultant cell is stimulated and crosslinked with formaldehyde or other crosslinkers, if necessary. (iv) The cell is lysed, and DNA is fragmented by sonication or other methods. (v) The complexes including the exogenous DB are immunoprecipitated with an antibody against the tag or isolated by other affinity purification procedures. (vi) The isolated complexes retain molecules interacting with the genomic region of interest. Subsequent purification of DNA, RNA, proteins, or other molecules allow their identification and characterization (Figure 4(c)).

**Figure 5:**
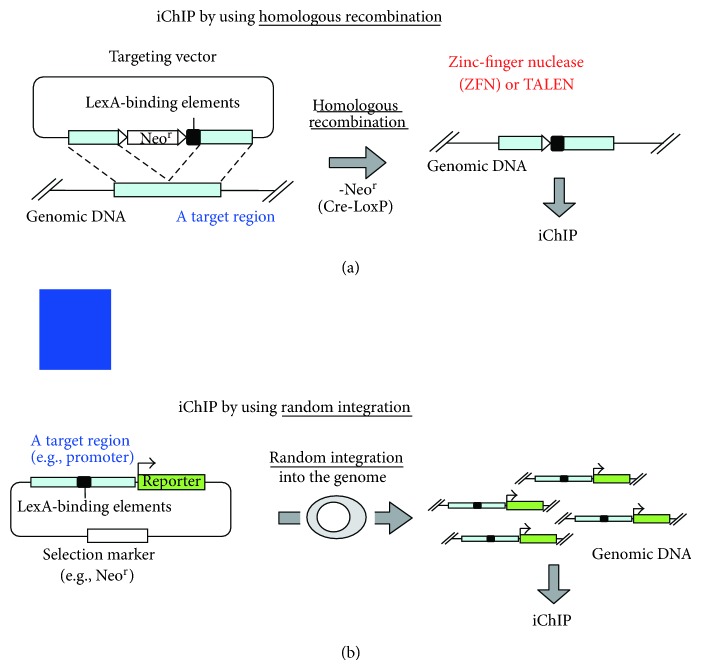
Knock-in and transgenic approaches of iChIP. (a) Knocking-in of LexA BE into the endogenous locus or (b) random integration of transgenes retaining LexA BE can be used for iChIP. Zinc-finger nucleases (ZFN) and TALEN technology make gene targeting much easier in cultured cell lines.

Knocking-in of LexA-binding elements (LexA BE) in the endogenous locus as well as transgene approach can be used for iChIP (Figure 5). Obviously, targeting an endogenous locus would be more physiological (Figure 5(a)). In contrast, when the transgene is known to harbor critical regulatory elements, random integration of transgenes retaining LexA BE (Figure 5(b)) would be beneficial because of potential increase in copy numbers, which makes biochemical analyses much easier. Thus, iChIP is a comprehensive approach to purify specific genomic regions of interest to identify interacting molecules including genomic DNA, proteins, RNAs, and others, with an emphasis on nonbiased search using next-generation sequencing (NGS), microarrays, and mass spectrometry (MS).

iChIP has two precursory technologies as its origins. Obviously, one is ChIP as described above. The other is locus-tagging with recognition elements of DNA-binding proteins. This technique has been widely used in live imaging of specific loci (reviewed in [27]). In addition, locus-tagging has also been used for biochemical purification of specific genomic regions in yeast [6]. Since genomic DNA is too large to be isolated, some measures are needed to make the target regions short enough for purification. To this end, specific genomic regions were excised by using the Cre-*lox*P system. The use of Cre-*lox*P system circularizes the floxed genomic regions suitable for biochemical purification. However, Cre-mediated circularization may break interaction between the target loci and interacting genomic regions. In addition, Cre-mediated circularization could not be used for crosslinked chromatin. Thus, this approach cannot be used when endogenous conformation is important such as detection of interchromosomal interaction.

### 3.2. Characteristics of**  **iChIP

iChIP has many advantages over other nonbiased search methods described above (Table 1). (i) iChIP enables us to perform nonbiased search for molecules interacting with specific genomic regions. (ii) Intergenomic interaction can be detected. It has not been shown whether PICh can be used for these analyses. In addition, “interaction” detected by 3C-based approaches does not necessarily mean physical interaction. In other words, since efficiency of enzymatic digestion is affected by locus accessibility, signal derived from 3C-based approaches may represent accessibility of the loci. In this regard, since iChIP can be performed without any enzymatic processes, detected signals represent physical interactions. (iii) iChIP has been used for detection of proteins and RNA interacting with the genome. In contrast, detection of interacting proteins and RNA is not feasible by 3C because they are removed in the procedure. It has not been shown whether PICh can be used for detection of interacting RNA. (iv) iChIP can be performed without any enzymatic reactions that may give rise to noise or artifactual signals. (v) Low-copy number loci can be analyzed by iChIP. In fact, we succeeded in identifying proteins interacting with a single endogenous locus (manuscript in preparation. See below). In contrast, application of PICh to low-copy number loci may be difficult as described above. (vi) Allele-specific analysis is feasible with iChIP because a specific allele can be tagged.

Although iChIP has many advantages over other techniques as described above, it has some disadvantages. (i) It requires generation of cells for iChIP analysis, that is, insertion of LexA BE into the target loci and expression of a tagged LexA DB. In this regard, knocking-in into the genome of cell lines has been more difficult than that of mouse embryonic stem cells. However, advent of zinc-finger nucleases (ZFN) [28] and TALEN technology [29] makes gene targeting much easier in cultured cell lines (Figure 5(a)). (ii) Insertion of LexA BE may affect chromatin structure such as nucleosome positioning and abrogate normal genome activities such as gene expression. Although the effects of insertion need to be tested empirically for each locus, we have guidelines to avoid potential aberrant effects caused by insertion of LexA BE. (a) For analysis of promoter regions near transcription start sites (TSSs), the insertion site should be several hundred base 5′ to the TSS so that the insertion would not inhibit transcription or disrupt nucleosome positioning. In contrast, for identification of binding molecules of genomic regions with distinct boundaries such as enhancers or silencers, the LexA BE can be directly juxtaposed to the regions because it is less probable that the insertion of LexA BE might inhibit their function. (b) The insertion site should not be conserved among species because conserved regions are often binding sites of essential binding molecules.

## 4. Application of iChIP

### 4.1. Identification of Proteins and RNA Interacting with Insulator

We applied iChIP to direct identification of components of insulator complexes, which function as boundaries of chromatin domains [30]. By combining iChIP with MS (iChIP-MS) and RT-PCR (iChIP-RT-PCR), we found that the chicken *β*-globin HS4 (cHS4) insulator complex contains an RNA helicase protein, p68/DDX5; an RNA species, steroid receptor RNA activator 1 (SRA1); and a nuclear matrix protein, Matrin-3, *in vivo*. Binding of p68 and Matrin-3 to the cHS4 insulator core sequence was mediated by CCCTC-binding factor (CTCF). Thus, our results showed for the first time that it is feasible to directly identify proteins and RNA bound to a specific genomic region *in vivo* by using iChIP.

The fact that p68/DDX5 was directly identified as an insulator component by iChIP clearly shows the power of iChIP. It took only several months for us to identify p68/DDX5 since the project was started. In contrast, it took more than ten years to identify p68/DDX5 as an insulator component by using conventional methods [31, 32] since the insulator was first discovered [33]. Thus, iChIP can accelerate the process of identification of components of chromatin complexes by 10–100-fold.

We also successfully detected SRA1 RNA [32] in the purified cHS4 insulator complex. Combination of iChIP with microarray or RNA-seq would be promising for nonbiased search for RNA associated with specific genomic regions, which cannot be achieved by other methods.

### 4.2. Detection of Genomic Interactions

It is of note that a pioneering work used locus-tagging for detecting interaction of specific genomic loci by genomic PCR [34]. After the initial publication of iChIP, iChIP has been used to detect genomic interaction in budding yeast in a nonbiased manner. Genome-wide iChIP studies were performed to find that pheromone-response genes regulated by a transcription factor, Ste12, have increased interchromosomal interactions in cells lacking Dig1 protein, a inhibitor of Ste12 [35]. They found that the increase in interchromosomal interactions is the basis of increase in intrinsic and extrinsic noise in the transcriptional outputs of the mating pathway. Thus, iChIP is a powerful technique to detect genomic interactions.

We are now attempting genome-wide search for genomic regions interacting with an endogenous locus by combining iChIP and NGS (iChIP-Seq). Preliminary results showed that iChIP-Seq is able to detect long-range genomic interactions such as interchromosomal interaction without ambiguity (manuscript in preparation), suggesting that it is useful for genome-wide identification of interacting genomic regions.

## 5. Future Application of iChIP

We have been optimizing experimental conditions of iChIP including development of a second generation 3xFLAG-tagged LexA DB, 3xFNLDD [36]. iChIP using 3xFNLDD was able to consistently isolate more than 10% of input genomic DNA, several-fold more efficient than the first-generation tagged LexA DB. In addition, elution conditions with 3xFLAG peptide have been optimized.

To increase the utility of iChIP, we are attempting to purify molecular complexes associated with an endogenous locus of higher eukaryotes. In this study, the LexA BE-inserted promoter region of the *Pax5* gene, which encodes the master lineage commitment transcription factor for B cell development, is purified by iChIP and subjected to MS. The *Pax5* gene is on the Z chromosome in the chicken, and the chicken mature B-cell line, DT40, used in the study has one Z chromosome. We identified multiple proteins interacting with the *Pax5* promoter. The identified proteins included transcription factors, DNA-binding proteins, histones, and other proteins potentially involved in transcriptional regulation (manuscript in preparation).

Combination of iChIP with SILAC (stable isotope labeling using amino acids in cell culture) [37] or iTRAQ (isobaric tag for relative and absolute quantification) [38] would be promising in comparing samples prepared in different conditions, for example, different cell types, in the absence or presence of stimulation, and so forth. In fact, we have been successfully identifying proteins associated with the *Pax5* promoter region for the above-mentioned DT40-derived cells using iChIP-SILAC (manuscript in preparation). Recently, application of iChIP-SILAC to yeast cells was reported [39].

Another important direction of application of iChIP is to detect novel epigenetic marks such as histone modifications. It has been shown that various chemical modifications on histones play crucial roles in genomic processes such as DNA replication, DNA repair, transcription, heterochromatinization by binding specific factors that, in turn, serve to alter the structural properties of chromatin [40]. Recent advancement of MS has enabled to identify novel histone modifications in a large scale [41]. In this regard, since distribution of some important epigenetic marks can be restricted in certain genomic domains, these marks can be missed due to dilution when whole genome is used as the source for MS. In contrast, since iChIP can purify specific genomic regions, it is possible to identify such rare epigenetic marks concentrated in those genomic regions.

Application of iChIP is not restricted to cultured cell lines but easily extended to organisms *in vivo*. In fact, iChIP was recently applied to cells of entire body of fruit fly [42]. We and our collaborators are now applying iChIP to mice by using knocking-in of LexA BE in ES cells and transgenic expression of a tagged LexA in transgenic mice.

Taken together, iChIP will be a powerful and useful tool to dissect “interactome” of a given genomic loci.

## Figures and Tables

**Table 1:** Comparison chart of nonbiased methods to analyze specific genomic regions.

Method	Non-biased analysis	DNA analysis	Protein analysis	RNA analysis	Include enzyme reactions	Low-copy number genes	Need transgenic	Allele-specific analysis
iChIP	Yes	Feasible	Feasible	Feasible	No	Feasible	Yes	Feasible
3C	Yes	Feasible	Not feasible	Not feasible	Yes	Feasible	No	Not feasible
PICh	Yes	Not reported	Feasible (telomeres)	Not reported	No	Not reported	No	Not feasible

## Abbreviations

iChIP: Insertional chromatin immunoprecipitation
ChIP: Chromatin immunoprecipitation
3C: Chromosome conformation capture
PICh: Proteomics of isolated chromatin
FISH: Fluorescent *in situ* hybridization
MS: Mass spectrometry.

### Glossary

Epigenetics: The research field of heritable changes in gene expression or cellular phenotype caused by mechanisms other than changes in the DNA sequences
Chromatin: The complexes of DNA sequences and interacting RNA and proteins in the nucleus.
